# The Effects of Equine-Assisted Learning on Adolescents with Internet Gaming Disorder

**DOI:** 10.3390/healthcare12030311

**Published:** 2024-01-25

**Authors:** Hyoungjin Park, Taewoon Jung

**Affiliations:** 1 School of Sports and Health, Kyungsung University, Busan 48434, Republic of Korea; hjpark79@ks.ac.kr; 2 Department of Special Physical Education, Yongin University, Yongin 17092, Republic of Korea

**Keywords:** equine-assisted learning, internet gaming disorder, gaming addiction

## Abstract

During the COVID-19 pandemic, internet gaming became more popular as a way to cope with stress, but excessive gaming can lead to mental health issues like internet gaming disorder (IGD). IGD has serious consequences, especially among children and young adults, and the gaming industry’s profits continue to grow. This study aims to understand the mental and behavioral health of adolescents with IGD participating in an equine-assisted learning (EAL) program and assess the changes in their addiction tendencies and emotional and behavioral problems. The results showed that the participants’ tendency towards internet gaming addiction and emotional and behavioral problems decreased immediately after the EAL program, but they rose again a month later. This suggests the importance of ongoing program involvement. As COVID-19 restrictions ease worldwide, this study highlights the increasing risk of IGD. It suggests that EAL could be a valuable approach to treating behavioral addictions, including gaming addiction. While prior research has shown the effectiveness of EAL in treating substance addiction, more research is needed to explore its potential in treating various types of addictions, such as gambling or gaming addictions.

## 1. Introduction

Stressful everyday situations often lead to a strong desire to escape, with many turning to internet gaming as a coping mechanism [1,2]. Since the COVID-19 pandemic and its ensuing physical distancing measures, internet gaming’s popularity has surged as a stress relief [3]. However, excessive gaming can adversely affect mental health, leading to internet gaming disorder (IGD) [4]. This disorder, marked by isolation, a loss of interest in other activities, and persistent gaming, despite harmful consequences, has particularly increased among children and adolescents during the pandemic due to remote schooling and limited outdoor activities [3,4,5,6]. Furthermore, with the growing interest in internet games, the gaming industry’s profits continue to grow and are estimated to exceed USD 200 billion globally by 2023 [7]. Therefore, IGD may become even more frequent among this vulnerable population, necessitating a prompt intervention [8]. Internet gaming disorder (IGD) was added to Section 3 of the Diagnostic and Statistical Manual of Mental Disorders (DSM-5) [9] in 2013 and is described by nine criteria, including preoccupation, tolerance, withdrawal, persistence, escape, problems, deception, displacement, and conflict [10]. IGD impairs daily living and academic performance, disrupts family relationships, impairs emotional development, and is associated with aggressive/oppositional behavior, violence, anxiety, and depression [9,11,12,13]. In addition, certain risk factors have been found common in pathological internet gamers, such as insecure attachment styles, familial cohesiveness, emotional dysregulation, poor social competence, aggression, increased impulsivity, loneliness, social inhibition, reduced self-control, and low self-esteem [3,6,7,8,11,12,14,15].

There are only a few specific methods, such as cognitive behavioral therapy (CBT), electroacupuncture (EA), solution-focused approaches, and medication interventions, available to alleviate the symptoms of IGD [16]. This study intends to acquire a better understanding of the mental and behavioral health statuses of adolescents with IGD who participated in an EAL program, as well as to test for changes in their propensity for internet gaming addiction and emotional and behavioral problems. The rationale behind using equine-assisted learning (EAL) for this population in the current study is that equine-assisted activities are suggested to improve adolescents’ levels of physical, emotional, psychological, and social problems [17,18,19,20]. Additionally, to date, no one has applied EAL to adolescents with IGD. EAL offers a unique therapeutic environment, distinct from traditional clinical settings. The interaction with horses in EAL can foster emotional growth and learning, as horses naturally provide immediate, non-judgmental feedback. This can help adolescents develop self-awareness, empathy, and social skills, which are often impaired in those suffering from IGD.

Moreover, EAL’s physical and outdoor nature can be a refreshing change for individuals accustomed to the sedentary and indoor nature of excessive gaming. Utilizing EAL for IGD also opens new avenues in therapeutic approaches, encouraging a broader exploration of alternative and complementary methods in mental health treatment, especially for conditions where conventional therapies have limitations or need augmentation. The novel application of EAL to adolescents with IGD could provide valuable insights into its potential as a versatile and effective therapeutic tool. We used EAL for adolescents with IGD and investigated its usefulness by analyzing participants’ changes in the propensity for internet gaming addiction and emotional and behavioral issues.

### 1.1. The Usefulness of EAL for Individuals with IGD

Horses are said to have benefits such as being non-judgmental and motivating [21]. They are helpful, as a metaphor [22], for developing self-esteem and self-efficacy [23,24,25], confidence [26], positive behavior [27], and are also effective in fostering trust and attachment [21]. Therefore, horses have been used in a variety of therapeutic service contexts, such as learning and counselling programs in correctional facilities [28], mental health facilities [26], and social services [29], as well as with high-risk youths and veterans who have experienced emotional and behavioral trauma [30,31].

EAL is a type of equine-assisted service (EAS) that uses a learning-based program to assist individuals in dealing with personal, emotional, and behavioral issues [15]. EAL is a group-led educational program that includes opportunities for individual attention throughout each session [32]. EAL involves organized, facilitator-led sessions with ongoing participant experience-related feedback. The group sessions allow participants to become involved in circumstances that need interactions with both the horse and other group members [33]. Therefore, EAL offers practical learning through mounted and unmounted activities to improve participants’ social functioning, self-awareness, understanding, empathy, accomplishment, confidence through problem-solving, and interpersonal skills [32]. According to previous studies, EAL was effective in improving attachment, confidence, communication, empathy, and social skills in adolescents [30], encouraging hope and decreasing depression in at-risk youths [19], and decreasing trauma symptoms [31,33,34], as well as growing self-control, greater family intimacy, and lesser impulsivity in adolescents with attention-deficit hyperactivity disorder (ADHD) [30].

### 1.2. Mental Issues of Adolescents with IGD

Research has reported statistically significant correlations between IGD and anxiety, stress, lower self-esteem, impulsiveness, loneliness, antisocial behavior, higher levels of aggression, anger control problems, and hyperactivity/inattention among adolescents [10,35,36,37]. In particular, depression has been identified as a risk factor for internet-related behaviors [38,39]. Previous studies have concluded that depression is linked to the onset and persistency of IGD in adolescents [40]. In particular, individuals with depression have a propensity to self-medicate by using the internet excessively, which is an avoidant coping mechanism that exacerbates rather than resolves real-life issues [41]. Due to the disruption of actual social interactions, IGD itself may also cause an increase in depressive symptoms, and this leads to social isolation and interpersonal issues, causing a vicious cycle [42,43].

### 1.3. Hypothesis

Based on previous studies, we hypothesized that EAL could reduce internet gaming addiction in adolescents with IGD. In addition, EAL could improve emotional and behavioral issues. Therefore, this study aimed to investigate the effect of EAL on internet gaming addiction and emotional and behavioral issues in adolescents with IGD.

## 2. Methods

### 2.1. Study Design and Participants

This study utilized a non-randomized pre- and post-test design with a single treatment group. Recruitment flyers were distributed to public officials in Korea with expertise in internet gaming addiction, with the assistance of the National Information Society Agency. Through the advertisement, twenty-seven adolescents aged between 11 and 15 years old and diagnosed with internet gaming disorder (IGD) were recruited. The demographic characteristics of the participants are presented in Table 1. The research protocol received approval from the Institutional Review Board of Jeonbuk Horse Industrial Complex Center. Prior to the commencement of the study, the purpose and methods of the research were comprehensively explained, and a written informed consent was obtained from all participants.

### 2.2. Assessment

The participants’ performances on the Korean Youth Self-Report (K-YSR) and the Korean Scale for Internet Addiction were evaluated at three points: baseline, immediately after the completion of the equine-assisted learning (EAL) program, and one month after the conclusion of the EAL program. The questionnaires were distributed in printed form, sealed, and collected by the primary investigator for coding and analysis. The collected data were encrypted, securely stored, and only accessible to the researchers responsible for coding, analyzing, and writing the research documents.

#### 2.2.1. The Korean Youth Self-Report

The Korean Youth Self-Report (K-YSR) is a self-assessment scale for adolescents, adapted from the Youth Self-Report (YSR) [44], and has undergone standardization [45]. It consists of 112 items designed to identify problem behaviors and clinical diagnoses. The reliability of this assessment has been found to be good, with confirmed discriminant and concurrent validity [46]. In this study, the K-YSR was utilized to assess the emotional and behavioral problems of adolescents diagnosed with internet gaming disorder (IGD).

#### 2.2.2. The Korean Scale for Internet Addiction for the Diagnosis of Internet Gaming Disorder

The Korean Scale for Internet Addiction (K-scale) is a self-assessment questionnaire used to measure internet addiction [47]. The Likert scale ranges from 1 (“never”) to 4 (“always”), yielding total scores between 40 and 160. The K-scale has demonstrated excellent internal consistency, with Cronbach’s alpha values of 0.89 for elementary school students and 0.91 for middle school students [48]. Previous research has examined the diagnostic utility of the K-scale for diagnosing internet gaming disorder (IGD) according to the DSM-5 criteria. In a study involving 274 adolescents, the symptom subscales of the K-scale (disability in daily life, withdrawal, deviant behavior, and tolerance) exhibited a higher diagnostic accuracy, correctly identifying 60.0% to 88.5% of participants at a cutoff point of 60/61 [49]. These findings suggest that the subscales of the K-scale serve as a reliable diagnostic tool for internet gaming disorders.

### 2.3. The EAL Program

The EAL program we applied was established by a diverse research team associated with the Riding Healing Center at The Korea Racing Authority (KRA), and with the advice of the Professional Association of Therapeutic Horsemanship International’s (PATH Intl.) licensed international experts. The program was conducted as a week-long prospective trial. All of the EAL-related programs were group interventions, with a 2:3 ratio of instructors to adolescents. The training sessions were 180 min each and were held twice a day (once a day on the first day and the last day) for eight days. Training sessions adhered to the following schedule: 30 min to care for the horse, including feeding and grooming; 20 min for equipping and tacking up the horse with the instructor; 60 min of therapeutic riding; 30 min for feedback and cooling down; 20 min for equipping and tacking the horse with the instructor; and 20 min to care for the horse, including feeding and grooming. The 15 sessions of EAL consisted of the following activities: Session 1: interacting with horses. Session 2: general equestrian education I. Session 3: general equestrian education II. Session 4: mounting and dismounting/basic posture at the walk/learning walk and halt aids. Session 5: learning to hold the reins and change direction with six corns at the walk. Session 6: half-seat position using hands at the walk. Session 7: two points at the walk and a half seat using hands at the trot. Session 8: two points at the walk and a half seat without hands at the trot. Sessions 9 and 10: posting trot at the ring. Session 11: posting trot at the half arena. Sessions 12 and 13: sitting/posting trot at the ring. Sessions 14 and 15: sitting/posting trot at the half arena.

### 2.4. Statistical Analysis

In addition to a descriptive analysis of the demographic information of 27 program participants, we also ran a one-way repeated measures ANOVA to assess the effect of EAL and the possible time effect (the one-month post-point). The analysis of the assumptions required for the calculations revealed no violation of the assumption of sphericity (Mauchly’s test) for all variables. Therefore, we applied the sphericity-assumed condition. Also, all pairwise comparisons were Bonferroni-corrected. All quantitative analyses were performed using SPSS 22.0. The significance level for the study was set to *p* < 0.05.

## 3. Results

### 3.1. The Korean Scale for Internet Addiction Subscales

Table 2 reveals the ANOVA results for the main variables of interest from the Korean Scale for Internet Addiction. The repeated measures of the ANOVA determined that the mean scores for disability in daily life (F(2, 44) = 9.33, *p* < 0.001), withdrawal (F(2, 44) = 20.03, *p* < 0.001), deviant behavior, (F(2, 44) = 12.94, *p* < 0.001), tolerance (F(2, 44) = 14.33, *p* < 0.001), and the total of subscales (F(2, 44) = 28.83, *p* < 0.001) differed significantly across the three test points.

Table 3 shows the results of the pairwise comparisons from the Korean Scale for Internet Addiction. The post hoc pairwise comparisons using the Bonferroni correction showed statistically significant decreased scores in all domains between the baseline (pre-) and post-assessments (see Table 3). When comparing the post to the follow-up assessment, withdrawal (12.52 vs. 14.96, *p* = 0.04) and the total of the subscale (57.35 vs. 64.74, *p* = 0.008) scores increased and reached significance. In addition, there were statistically significant differences between the baseline (pre-) and the follow-up, except for the disability in daily life score. This means that the level of internet gaming addiction decreased after the EAL program and that, except for the disability in daily life score, there was no baseline level of internet gaming addiction in all areas after one-month post-assessment.

### 3.2. The Korean Youth Self-Report

Table 4 shows the results of the ANOVA for the main variables of interest from the Korean Youth Self-Report. The repeated measures ANOVA determined that the mean results of anxiousness/depression (F(2, 52) = 7.19, *p* = 0.002), withdrawal/depression (F(2, 52) = 12.61, *p* < 0.001), somatic complaints (F(2, 52) = 4.63, *p* = 0.01), social problems (F(2, 52) = 11.36, *p* < 0.001), thought problems (F(2, 52) = 18.02, *p* < 0.001), attention problems (F(2, 52) = 16.81, *p* < 0.001), rule-breaking behavior (F(2, 52) = 11.23, *p* < 0.001), and aggressive behavior (F(2, 52) = 11.31, *p* < 0.001), differed significantly across the three test points.

Table 5 presents the pairwise comparison results from the Korean Youth Self-Report. The post hoc pairwise comparisons using the Bonferroni correction showed statistically significant decreased scores in all domains between the baseline (pre-) and the post-assessment (see Table 5). However, when comparing the post-to-follow-up assessment, only thought problems and rule-breaking behavior were not statistically increased. Additionally, there was a statistically significant difference between the baseline (pre-) and the follow-up in thought problems (64.72 vs. 68.03, *p* = 0.002).

## 4. Discussion

The COVID-19 epidemic severely reduced people’s outside activities and face-to-face interactions. As people stay home longer, they spend more time participating in digital leisure activities, such as games [3,50]. It has been demonstrated that gaming can help people escape negative feelings and experience happy ones [51]. However, it can also enhance gaming addiction and have a negative influence on mental health [52]. Understandably, there is a need to alleviate such consequences of the pandemic, like IGD. The present results show the following:

1. The propensity for internet gaming disorder in our participants was statistically decreased after the EAL program;
2. Positive changes were observed in various emotional, physical, and behavioral problems after participating in the EAL program;
3. The positive changes that appeared immediately after participation in EAL returned to the level measured by baseline a month after the program was over.

An important finding was that the tendency for internet gaming addiction decreased immediately after participating in the EAL program. Before the EAL program, the participants’ internet gaming addiction score was 76 points, which was much higher than the cutoff score of 60 points. However, after participating in the program, the score was 57.35 points, which was outside of the internet gaming addiction level and fell into the normal range. This is a very encouraging result, but after one month of participating in the program, the score rose again to 64 points. Of course, the score of 64 is statistically lower than before participating in the EAL program. However, considering that it has risen to the level of addiction again, it also shows the need for continuous participation in the program. The disability in daily life in the Korean Scale for Internet Addiction subscales is related to the inability to lead a proper daily life due to excessive gaming time caused by an obsession with games. Gaming time is positively related to the experience of flow; the positive experience of flow can encourage more intense gaming and, therefore, the development of internet gaming disorder [52,53,54]. It can be assumed that, by participating in the EAL program, the flow formed through internet games can be changed into a flow for horse-related activities, including horseback riding, grooming, etc., for adolescents with internet gaming disorder. The results of withdrawal, deviant behavior, and tolerance in the Korean Scale for Internet Addiction subscales are related to the main symptoms shown by adolescents with internet gaming disorder. The IGD criteria refer to the withdrawal symptoms, including irritability, anxiety, or sadness, that follow the cessation of internet gaming [55]. Therefore, lowering the Korean Scale for Internet Addiction subscales’ scores means that the level of internet gaming disorder has improved. In line with the experimental research on IGD in adolescents [56], interventions resulted in a decrease in internet addiction after EAL. In the current study, EAL might have to be repeated for its positive effect.

In addition, changes in emotional, physical, and behavioral problems after participating in the EAL program were also essential findings of this study. Through the Korean Youth Self-Report, adolescents with internet gaming disorder showed statistically significant positive results in all areas, including anxiousness/depression, withdrawal, somatic complaints, social problems, thought problems, attention problems, rule-breaking behavior, and aggressive behavior after participating in the EAL program. The results of these emotional/behavioral corrections were similar to those of past studies, proving the validity of the hypothesis established by the researchers in this study. However, as shown in the results of internet gaming addiction changes, after one month of participating in the program, adolescents with internet gaming disorder again showed emotional/behavioral problems at the same level as the baseline in all areas except thought problems. This observation underscores the complexity of mental health disorders, especially those related to addictive behaviors like excessive internet gaming. It suggests that a multifaceted approach, possibly involving cognitive behavioral therapy, ongoing support, and strategies tailored to individual cognitive challenges, might be necessary for a more comprehensive and lasting improvement. Future prospective research in larger samples, involving longer periods of the EAL program is required to verify and expand upon current observations.

Physical distancing due to the COVID-19 pandemic is being abolished around the world, and the risk of COVID-19 is lower than before, but the side effects, such as an increase in IGD, are still appearing. IGD is a type of a behavioral addiction, and this study showed the potential for EAL to be effective in treating behavioral addictions. The effects of EAL on substance addictions, such as inhalant drugs in adolescents, have been shown in prior studies. However, there is currently no research on behavioral addictions, such as gambling or gaming addictions. Future research is needed to recognize that EAL can be used in the treatment of behavioral addictions and to verify what effects EAL has on various addictions. For example, suppose a horse-racing gambling addict is rehabilitated using horses. In that case, it is expected that there may be more favorable benefits and that it may be more effective in breaking the addiction.

## 5. Limitations

Some of the limitations of this study are the inability to provide the effectiveness of the equine-assisted learning (EAL) program across the different severity levels of internet gaming disorder (IGD); the confounding factors for participants who did not respond as promptly; the presence of comorbid mental health illnesses; the differences in response rates among the participants with a history of substance use or other forms of addiction; and the long-term effects of the EAL program. Due to the limited data available, it was challenging to thoroughly analyze and differentiate the responses of the EAL program based on the severity of IGD, identify specific demographic or lifestyle factors that might affect the program’s effectiveness, and assess comorbid conditions and their impact on the program’s outcomes. These limitations point to the need for future research with a broader scope of data collection and analysis to address these critical aspects. Such research could provide more comprehensive insights into the effectiveness of the EAL program for various IGD severity levels, the influence of comorbid conditions, and the program’s long-term impact on IGD.

## Figures and Tables

**Table 1:** Participant demographics.

Participant Characteristics	Age (yr)	Height (cm)	Mass (kg)	BMI (kg/m^2^)
Mean	12.52	154.41	46.33	19.45
Standard deviation	1.31	9.07	6.79	2.62

**Table 2:** Results of repeated measures ANOVA from the Korean Scale for Internet Addiction subscales.

	Baseline (pre)	Post	Follow-up (One-Month Post)	df, Error df	F	Sig.	*η_p_* ^2^
	M ± SD	M ± SD	M ± SD				
Disability in daily life	24.26 ± 5.65	20.30 ± 6.82	22.00 ± 5.29	2, 44	9.33	<0.001 *	0.30
Withdrawal	18.61 ± 4.02	12.52 ± 4.21	14.96 ± 4.58	2, 44	20.03	<0.001 *	0.48
Deviant behavior	16.74 ± 4.61	12.52 ± 4.05	14.00 ± 3.86	2, 44	12.94	<0.001 *	0.37
Tolerance	16.91 ± 3.54	12.00 ± 4.52	13.78 ± 4.15	2, 44	14.33	<0.001 *	0.39
Total	76.52 ± 2.90	57.35 ± 3.34	64.74 ± 3.04	2, 44	28.83	<0.001 *	0.57

Notes: M = mean; SD = standard deviation; df = degree of freedom; F = F-ratio; Sig. = significance; *η_p_*^2^ = partial eta squared; *: *p* < 0.05.

**Table 3:** Pairwise comparisons of assessment points from the Korean Scale for Internet Addiction subscales.

	Baseline (pre) vs. Post	Post vs. Follow-up	Baseline (pre) vs. Follow-up
	md, 95%*CI*, Sig.	md, 95%*CI*, Sig.	md, 95%*CI*, Sig.
Disability in daily life	3.96, (1.51, 6.40), 0.001 *	−1.70, (−3.74, 0.35), 0.79	2.26, (−0.36, 4.88), 0.11
Withdrawal	6.09, (3.34, 8.83), <0.001 *	−2.43, (−4.74, −0.13), 0.04 *	3.65, (1.19, 6.11), 0.003 *
Deviant behavior	4.22, (1.80, 6.63), 0.001 *	−1.48, (−3.67, 0.72), 0.29	2.74, (0.84, 4.63), 0.003 *
Tolerance	4.91, (2.21, 7.61), <0.001 *	−1.78, (−4.10, 0.54), 0.17	3.13, (0.96, 5.30), 0.003 *
Total	19.17, (11.72, 26.62), <0.001 *	−7.39, (−13.04, −1.74), 0.008 *	11.78, (5.21, 18.36), <0.001 *

Notes: md = mean difference; *CI* = confidence interval; *: *p* < 0.05.

**Table 4:** Results of repeated measures ANOVA from the Korean Youth Self-Report.

	Baseline (pre)	Post	Follow-up (One-Month Post)	df, Error df	F	Sig.	*η_p_* ^2^
	M ± SD	M ± SD	M ± SD				
Anxious/depressed	69.90 ± 14.59	61.01 ± 14.29	68.30 ± 12.48	2, 52	7.19	0.002 *	0.22
Withdrawn/depressed	68.84 ± 11.04	57.58 ± 12.52	63.59 ± 9.97	2, 52	12.61	<0.001 *	0.33
Somatic complaints	52.00 ± 7.31	48.80 ± 7.82	52.68 ± 8.01	2, 52	4.63	0.01 *	0.15
Social problems	65.69 ± 15.48	55.72 ± 13.79	63.26 ± 11.59	2, 52	11.36	<0.001 *	0.30
Thought problems	75.96 ± 12.01	64.72 ± 13.00	68.03 ± 12.08	2, 52	18.02	<0.001 *	0.41
Attention problems	57.13 ± 8.07	47.57 ± 10.34	54.14 ± 7.75	2, 52	16.81	<0.001 *	0.39
Rule-breaking behavior	81.36 ± 13.49	69.12 ± 12.86	75.18 ± 12.73	2, 52	11.23	<0.001 *	0.30
Aggressive behavior	71.43 ± 11.83	61.37 ± 13.36	68.52 ± 10.00	2, 52	11.31	<0.001 *	0.30
Internalizing	67.51 ± 11.50	57.87 ± 12.71	64.98 ± 10.63	2, 52	12.36	<0.001 *	0.32
Externalizing	78.45 ± 12.65	66.31 ± 13.58	73.66 ± 11.45	2, 52	12.97	<0.001 *	0.33
Total problems	79.90 ± 12.30	65.80 ± 15.55	74.67 ± 12.48	2, 52	18.80	<0.001 *	0.42

Notes: M = mean; SD = standard deviation; df = degree of freedom; F = F-ratio; Sig. = significance; *η_p_*^2^ = partial eta squared; *: *p* < 0.05.

**Table 5:** Pairwise comparisons of assessment points from the Korean Youth Self-Report.

	Baseline (pre) vs. Post	Post vs. Follow-up	Baseline (pre) vs. Follow-up
	md, 95%*CI*, Sig.	md, 95%*CI*, Sig.	md, 95%*CI*, Sig.
Anxious/depressed	8.89, (2.48, 15.29), 0.004 *	−7.28, (−12.53, −2.03), 0.004 *	1.60, (−5.74, 8.95), 1.00
Withdrawn/depressed	11.26, (5.84, 16.69), <0.001 *	−6.02, (−12.18, 0.15), 0.06 *	5.25, (−0.37, 10.87), 0.07
Somatic complaints	3.20, (0.23, 6.16), 0.03 *	−3.87, (−7.53, −0.21), 0.03 *	−0.67, (−4.43, 3.09), 1.00
Social problems	10.97, (5.78, 16.17), <0.001 *	−7.54, (−13.21, −1.88), 0.01 *	3.43, (−3.63, 10.49), 0.67
Thought problems	11.24, (7.08, 15.40), <0.001 *	−3.31, (−8.60, 1.99), 0.37	7.94, (2.70, 13.17), 0.002 *
Attention problems	9.56, (5.48, 13.63), <0.001 *	−6.57, (−10.69, −2.45), 0.001 *	2.99, (−1.74, 7.71), 0.35
Rule-breaking behavior	12.23, (6.51, 17.96), <0.001 *	−6.06, (−12.95, 0.83), 0.10	6.17, (−0.94, 13.29), 0.11
Aggressive behavior	10.05, (5.59, 14.52), <0.001 *	−7.14, (−12.94, −1.34), 0.01 *	2.91, (−3.36, 9.18), 0.74
Internalizing	9.64, (4.70, 14.58), <0.001 *	−7.11, (−11.96, −2.26), 0.003 *	2.53, (−3.08, 8.14), 0.779
Externalizing	12.15, (7.18, 17.11), <0.001 *	−7.35, (−13.70, −1.01), 0.019 *	4.79, (−2.17, 11.75), 0.27
Total problems	14.10, (9.04, 19.16), <0.001 *	−8.87, (−15.00, −2.74), 0.003 *	5.23, (−1.33, 11.78), 0.15

Notes: md = mean difference; *CI* = confidence interval; *: *p* < 0.05.

## Data Availability

The data presented in this study are available on request from the corresponding author.
